# Predicting Pressure Sensitivity to Luminophore Content and Paint Thickness of Pressure-Sensitive Paint Using Artificial Neural Network

**DOI:** 10.3390/s21155188

**Published:** 2021-07-30

**Authors:** Mitsugu Hasegawa, Daiki Kurihara, Yasuhiro Egami, Hirotaka Sakaue, Aleksandar Jemcov

**Affiliations:** 1 Department of Aerospace and Mechanical Engineering, University of Notre Dame, Notre Dame, IN 46556, USA; mhasegaw@nd.edu (M.H.); dkurihar@nd.edu (D.K.); hsakaue@nd.edu (H.S.); 2 Department of Mechanical Engineering, Aichi Institute of Technology, 1247 Yachigusa, Yakusa-cho, Toyota 470-0392, Aichi, Japan; egami@aitech.ac.jp

**Keywords:** pressure-sensitive paint, artificial neural network, machine learning, data augmentation

## Abstract

An artificial neural network (ANN) was constructed and trained for predicting pressure sensitivity using an experimental dataset consisting of luminophore content and paint thickness as chemical and physical inputs. A data augmentation technique was used to increase the number of data points based on the limited experimental observations. The prediction accuracy of the trained ANN was evaluated by using a metric, mean absolute percentage error. The ANN predicted pressure sensitivity to luminophore content and to paint thickness, within confidence intervals based on experimental errors. The present approach of applying ANN and the data augmentation has the potential to predict pressure-sensitive paint (PSP) characterizations that improve the performance of PSP for global surface pressure measurements.

## 1. Introduction

Pressure-sensitive paint (PSP) using a luminophore has been widely used as pressure sensor in fluid dynamics studies [1,2,3,4]. A key feature of PSP is the sensitivity to pressure variations [5,6,7,8,9,10,11]. The luminescence of the PSP is converted to pressure using the Stern–Volmer equation [11]. Given the relationship between the luminescence of the PSP and corresponding pressure, a PSP with higher pressure sensitivity is desirable.

The higher pressure sensitivity is obtained by adjusting the paint formulation of the PSP. The luminophore concentration of the PSP influences the pressure sensitivity [11,12,13]. Other components such as the thickness of the PSP applied to a surface also affect the pressure sensitivity [14,15,16,17]. The pressure sensitivity of the PSP is determined by an experimental calibration. Typically, one component of a PSP is varied at a time to extract the correlation between the component and pressure sensitivity. Depending on the range of required pressure sensitivity, an extensive range of the components sensitive to pressure sensitivity must be experimentally investigated. Such investigations require significant time since many experimental coupons must be created and calibrated. Ideally, all components must be investigated, including their mutual interactions, in order to map their influence on the pressure sensitivity of the PSP. Given the complex nature of the correlations between the pressure sensitivity and components, it is challenging to incorporate all of the components into a parametric study due to experimental time constraints. It is highly desirable to create a model linking the pressure sensitivity and components related to the PSP. In this work, we investigate the use of artificial neural networks (ANNs) to create the model.

ANNs are often used to approximate nonlinear functions with great success in various fields including chemical engineering [18,19,20], civil engineering [21,22], electrical engineering [23], computer engineering [24], and interdisciplinary engineering [25,26,27,28,29,30,31]. In this work, the ANN is applied to predictions of the pressure sensitivity of a PSP. The ANN architecture is constructed and trained using the PSP datasets measured. A practical challenge is that the prediction accuracy of the ANN depends on the number of data points [32]. Large datasets reduce overfitting and generalize the ANN predictive capabilities [32,33,34,35] However, due to the extensive time required for experiments, the number of data points measured is typically small, consisting of less than a hundred data points. Data augmentation techniques are often used to increase the number of data points [36]. 

In the present paper, we study the suitability of the use of an ANN in predicting the pressure sensitivity of the PSP to luminophore content and paint thickness. The ANN is used instead of typical approaches such as phenomenological modeling or statistical modeling approaches. The correlation among the three parameters is hard to obtain by the typical approaches because the phenomenon that determines pressure sensitivity is coupled by chemical and physical factors. As components increase, the application of typical approaches becomes increasingly difficult. The main goal of the present study is to enable the replacement of time-consuming and expensive experiments by an ANN trained to predict pressure sensitivity to given components related to PSP. To the best of our knowledge, the present study is the first attempt to apply an ANN to PSP development. The ANN is trained using an experimental dataset to obtain the correlation between pressure sensitivity with luminophore content and paint thickness. The training dataset includes the following variables: paint thickness (mm), luminophore content (mg), and pressure sensitivity (%/kPa). This paper also investigates the general applicability of the ANN trained using an augmented dataset utilizing experimental errors. The data augmentation technique is used to increase the number of data points from experimental observations.

## 2. Experimental and Augmented Dataset

An experimental dataset was used to train an ANN and evaluate the prediction performance of the trained ANN. The experimental dataset was collected through the characterization of PSP coupons. Luminophore content and paint thickness are mutually independent variables, and they are chemical and physical factors that impact pressure sensitivity, respectively. The luminophore content and paint thickness of PSP coupons were selected to design a PSP that increases the pressure sensitivity as much as possible. The pressure sensitivity was obtained for different luminophore contents and paint thicknesses. The experimental dataset, D_O_, consisting of a total of 84 measurements, was split into 90% for the training dataset; D_O,76_, composed of 76 data points and 10% for the test dataset; and D_O,8_, consisting of 8 data points. Typically, 80% and 20% of data points or 90% and 10% of data points are used for training and testing, respectively. The present study selected the latter ratio to increase the training dataset because the available experimental data is limited to less than 100 points. Table 1 shows the range and the experimental errors of the experimental dataset for PSP coupons. Details of the collection of experimental datasets are described in Appendix A.

**Figure 1:**
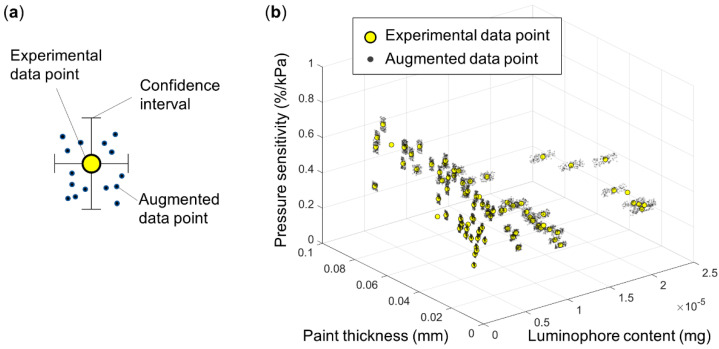
(**a**) Schematic concept of data augmentation by newly produced data points (augmented data points) from experimental data points using confidence intervals based on the experimental errors. (**b**) An example of data augmentation: 100 augmented data points, D_A,7600_, were created around each experimental data point, and a total of 76 × 100 was produced as output for pressure sensitivity for different paint thicknesses and luminophore contents as inputs.

The augmented dataset, D_A_, was used to train the ANN. The augmented dataset was produced using the experimental dataset. Figure 1a shows the schematic concept of the data augmentation used in the present study. The data augmentation produced the data points distributed within the confidence intervals based on experimental errors at each observation point. Here, it is assumed that there is no correlation between the error and the variable (i.e., all variables are mutually independent). The randomness of the dataset produced by the data augmentation follows a Gaussian distribution centered at zero. The data augmentation produced data, D_A,n_, consisting of 76, 760, 7600, 76,000, and 760,000 entries starting from the training dataset, D_O,76_, with 76 data points. The subscript n in D_A,n_, indicates the total number of entries obtained through the augmentation procedure. The number of augmented data points was varied to find the one that yields the most accurate ANN model. Figure 1(b) shows an example of the augmented dataset, D_A,7600_, with 7600 data points created around each observation point. The data augmentation process is repeated for each experimentally observed data point corresponding to various paint thicknesses and luminophore contents.

Dataset standardization was performed to avoid numerical issues and avoid divergence of the training process during the training caused by different input scales in a dataset with differing physical units and values [37]. Standardization of the dataset was achieved by normalization using the mean and standard deviation. 

## 3. ANN Model Development

### 3.1. Architecture of ANN Models

**Figure 2:**
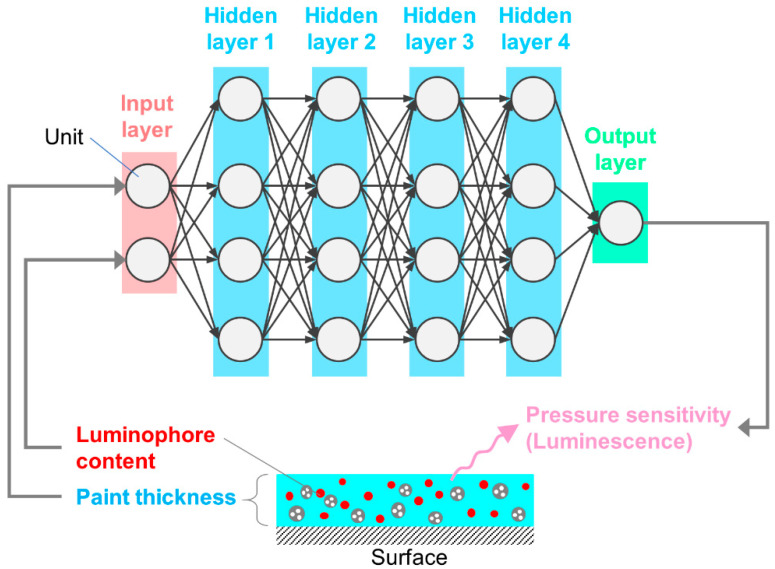
Architecture and corresponding dataset of the artificial neural network (ANN) model for predicting pressure sensitivity to luminophore content and paint thickness.

Figure 2 shows the schematic description of the architecture of the ANN model used in the present study. The architecture was realized through a fully connected, deep ANN consisting of the input layer with two units, the hidden layers with four units, and the output layer with a single unit. Here, units are also called neurons in terms of a biological brain. Luminophore content and paint thickness are considered to constitute inputs of the ANN models, while the pressure sensitivity constituted an output of the ANN models. The activation function, rectified linear unit (ReLU), was used in the hidden layers [38]. Further details regarding the architecture and studies used to define the final architecture are provided in Appendix B. The selected architecture of the ANN model is summarized in Figure 2.

### 3.2. Training of ANN Models

ANN models were trained using the augmented datasets, D_A,n_. An ANN model was also trained using the experimental training dataset, D_O,76_, in order to compare with the ANN models trained by augmented datasets. The ANN models were trained for 40,000 epochs. The ANN model weights were optimized using the Adam optimizer [39]. Because the random selection of the initial ANN model weights in the training influences updated weights, resulting in different predictions, each ANN model was trained 10 times to obtain the median. The learning rate was set at 10^−3^. Table 2 summarizes the ANN models for different training datasets.

## 4. Evaluation of the Prediction Performance of Trained ANN Models

The performance of the trained ANN models was assessed by the accuracy of the prediction of the ANN models when the test dataset, D_O,8_, was used. A metric, mean absolute percentage error (MAPE), was used in quantitatively assessing the accuracy of the predictions [40,41]. The MAPE was calculated using the following equation [40,42]:

(1) $$\mathrm{MAPE}\%=\frac{1}{N}\sum_{i=1}^{N}\frac{\left|o_{i}-p_{i}\right|}{o_{i}}\times 100.$$

Here, *o_i_* is the vector of observed values, *p_i_* is the vector of predicted values, *i* is the index, and *N* is the number of data points.

## 5. Results and Discussion

**Figure 3:**
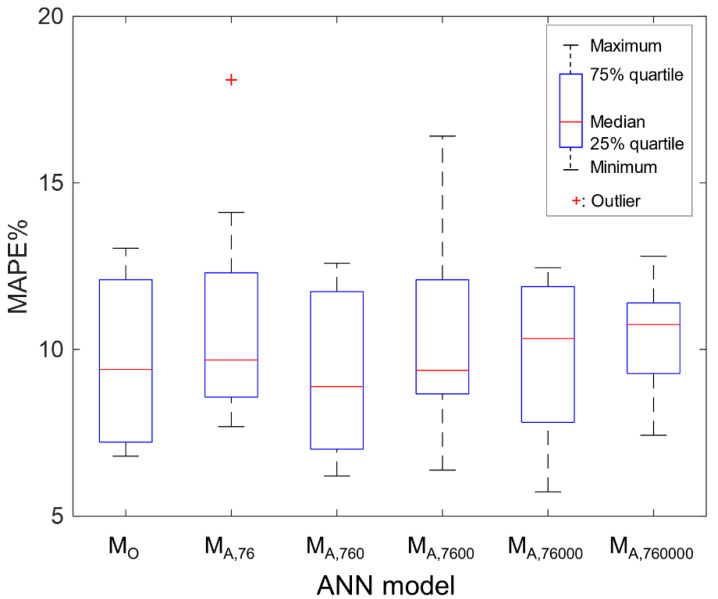
Comparison of mean absolute percentage error (MAPE) for different ANN models. Training and testing were repeated 10 times for each ANN model. The figure indicates that M_A,760_ has the lowest MAPE.

Figure 3 shows the comparison of the MAPE obtained in testing for different ANN models. All models use the same architecture (i.e., the size of layer and unit is the same for all models). However, each model was trained using a different training dataset, as shown in Table 2, and MAPE was computed in the testing phase. The procedure of obtaining the MAPE (for example, M_A,760_, shown in Figure 3) is as follows: (1) the architecture is trained using a training dataset of 760 points; (2) prediction is obtained using the test data of 8 points; (3) MAPE is computed; (4) step (1) to (3) is repeated 10 times. Maximum, minimum, 75% quartile, 25% quartile, and median of MAPE are presented in the boxplot. MAPE is a measure of the mean of the absolute error relative to the test datasets. MAPE scales the overall accuracy of the prediction through the entire domain. The lower the MAPE values, the higher the accuracy of the prediction. M_A,760_ showed the lowest median MAPE of 8.9% in ANN models using the augmented datasets. As the number of augmented data points increased, the median MAPE increased where larger augmented data points than M _A,760_ were used, as shown in M_A,7600_, M_A,76000_, and M_A,760000_. By comparing all M_A,n_ models trained by using augmented datasets, it was found that the number of augmented data points influences the MAPE. It was also found that an investigation of the effect of the number of augmented points is required to minimize the MAPE. M_O_ showed a median MAPE of 9.4%, which was larger than that of the MAPE of 8.9% for M_A,760_. By comparing M_O_ with all M_A,n_ models, it is found that the use of augmented datasets minimizes the MAPE more than that of M_O_. The augmentation achieved greater accuracy. In the present study, the augmented data set consisting of 760 points is considered sufficient to lower the MAPE in the testing. 

**Figure 4:**
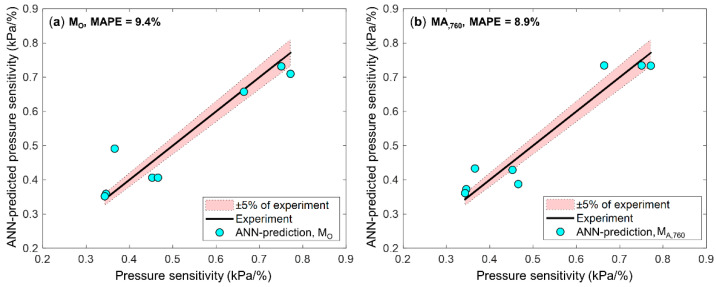
Prediction accuracy of M_O_ and M_A,760_ models for the test dataset. The shaded area enclosed with a dashed line indicates the confidence interval of ±5% of the experimental measurement. (**a**) The model M_O_, trained by D_O,76_. Four of eight test points were within 5% of the experimental measurement and MAPE was 9.4%. (**b**) The model M_A,760_, trained by augmented dataset, D_A,760_. Four of eight test points were within 5% of the experimental measurement and MAPE was 8.9%. The figure indicates that both models predict the pressure sensitivity within ±5% of the experimental measurement.

Figure 4 shows the accuracy of pressure sensitivity predicted by M_O_ and M_A,760_ models when MAPE was the median of the MAPE values for those models. The confidence interval based on the experimental error of pressure sensitivity (i.e., ±5%) was selected as an interval to evaluate how precisely the ANN approximates the pressure sensitivity. By comparing the relative error of M_O_ with M_A_, it was shown that four of eight test points were within 5% of the experimental measurement for both models. This indicates that both models predict the pressure sensitivity within ±5% of experimental measurement, while M_A_ showed the more accurate and lower MAPE than M_O,_ as shown in the comparison of the MAPE in the previous section.

## 6. Conclusions

Reducing time-consuming and expensive experiments is essential to enhancing PSP development. The present study investigated the application of an ANN to predict pressure sensitivity of the PSP to luminophore content and paint thickness with fewer than 10^2^ data points. The ANN model was built and trained on the dataset produced by a data augmentation technique based on the experimental errors. It was concluded that the ANN model can obtain the correlation between pressure sensitivity with luminophore content and paint thickness in PSP development. The ANN model will reduce experimental costs. The ANN models trained by augmented datasets achieved 8.9% of MAPE in predicting pressure sensitivity_._ Augmented datasets have the potential to reduce MAPE with a small number of experimental data points.

## Figures and Tables

**Table 1:** Range and experimental errors of experimental dataset for pressure-sensitive paint (PSP) coupons.

Component	Minimum	Maximum	Relative Error
Paint Thickness (µm)	4.532	67.635	±9% of paint thickness
Luminophore Content (mg)	3.0 × 10^−8^	2.0 × 10^−5^	±7% of luminophore content
Pressure Sensitivity (%/kPa)	0.2873	0.8094	±5% of pressure sensitivity

**Table 2:** ANN models. D_O,n_ represents an experimental dataset and D_A,n_ represents an augmented dataset. n is the number of data points.

ANN Model	Training Dataset	Test Dataset
M_A,76_	D_A,76_	D_O,8_
M_A,760_	D_A,760_	D_O,8_
M_A,7600_	D_A,7600_	D_O,8_
M_A,76000_	D_A,76000_	D_O,8_
M_A,760000_	D_A,760000_	D_O,8_
M_O_	D_O,76_	D_O,8_

**Figure A1:**
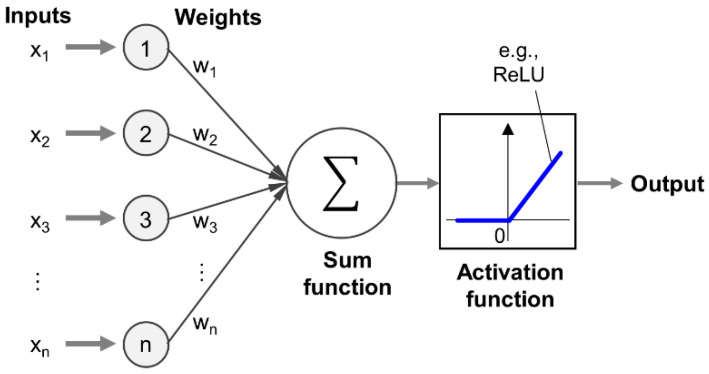
Simple neural network. ReLU: rectified linear unit.

## Data Availability

The data presented in this study are available on request from the corresponding author, upon reasonable request.

## Appendix A

This section presents the process of designing PSP where the experimental dataset was collected. Characterization of PSP was experimentally performed to design a PSP that increases pressure sensitivity as much as possible. A temperature/pressure-controlled chamber was used to place a PSP coupon under a controlled environment. Excitation and emission spectra of the PSP coupon were measured using a spectrometer. Luminescent intensity, temperature sensitivity, and pressure sensitivity (i.e., functions of PSP) were characterized. Luminophore concentration, luminophore content, paint thickness, and binder volume (i.e., components of PSP) were varied in order to identify a component that influences pressure sensitivity. To reduce experimental errors in the measurement caused by factors that vary from one measurement to another, the measurement was repeated multiple times.

## Appendix B

An ANN is a mathematical description of the nervous system’s structure in the human brain [43]. The simplest ANN (i.e., a simple perceptron) consists of an input layer and output layers of nodes. Those nodes are mutually fully connected. The perceptron is built in multiple layers consisting of multiple connected nodes. An output is produced through the ANN when a signal (i.e., data) is given to the input layer. The inside of the ANN contains weights to produce the desired output (i.e., prediction) corresponding to a given input. Figure A1 shows a simple ANN that consists of inputs, output, and inside the ANN, including weights, biases, a sum function, and activation function. Input is given from another system. The weight is a value that represents the influence of the previous layer’s input set or another processing element on this process element. The sum function calculates the net effects of inputs and weights on this process element (i.e., the weighted sum of nodes). The weighted sum contains a coefficient, w, as the weight, and a constant term called a bias. The activation function is used to determine the output value from the weighted sum and introduce a nonlinear effect into the output. The rectified linear unit (ReLU) is typically used as an activation function for nonlinear regression analysis [38].

Predictions using an ANN require training, as ANN architecture learns from the training dataset. By the back-propagation method [44], a constructed ANN architecture is trained to determine the weights and biases of the ANN that minimize the error between the ANN output (i.e., predicted values) and training dataset (i.e., observed values). A loss function is used to quantitatively evaluate the error. While various loss functions are available in ANN training, mean squared error (MSE) is commonly used to minimize the L2 norm of the error. The gradient descent method is used to find the weight and bias that minimize the error between the ANN output and training dataset [45]. The gradients of the loss function with respect to each weight and bias are calculated using the chain rule and partial derivatives. The gradients determine the updated directions of the weight and bias that reduce the loss function. The gradient descent method commonly used is Adam [39]. The weights are updated until the partial derivative of the loss function is zero during training. The amount that the weights are updated is called the learning rate. A larger learning rate yields faster convergence of the training; however, the training could be unstable. A lower learning rate stabilizes the training but requires a large number of iterations to converge. The trained ANN is used as an ANN model to make predictions based on a given input. The prediction performance of the ANN model is evaluated using metrics such as MSE, MAE, MAPE, *R*^2^, and RMSE, depending on the focus of the assessment. Further details on ANN are reviewed in references [26,46,47,48,49].

Construction of ANN architecture is required to develop an ANN model for prediction. A parametric study was preliminarily performed to determine the ANN architecture (i.e., size of units and layers) used in the present study. An ANN was constructed for different sizes of units and layers. The size of units and layers were varied to find a suitable ANN architecture that minimizes error between the prediction and training dataset, error’s bias, and error’s variance. ANN was trained using the Keras Regressor, an open-source ANN library, running with the TensorFlow, an open-source ANN library, under the Python environment, a programming language. The augmented dataset described in Section 2 was used for training. MSE was used as a loss function for training. MSE is calculated using the following equation:

(A1) $$\mathrm{MSE}=\frac{1}{N}\sum_{i=1}^{N}{\left(o_{i}-p_{i}\right)}^{2}.$$

The variance of MSE was also assessed because a lower value of the MSE does not indicate that the precision in predictions is always better in the generalization that captures the dominant trend between inputs and outputs and fits the predictions within that trend [32].

ANN was trained using an augmented dataset of 100, 1000, and 10,000 entries. Table A1, Table A2 and Table A3 shows the MSE of the ANN for different sizes of layers and units. Table A4, Table A5 and Table A6 shows the variance of the MSE of the ANN for different sizes of layers and units. The size of hidden layers was varied from 1 to 5. The unit in the hidden layer was varied from 2, 4, 8, and 16. The criteria to determine ANN was as follows: (1) MSE is less than 0.1; (2) variance is as small as possible. The size of the hidden layer was determined to be 4, and the unit size was determined to be 4 based on the criteria.

**Table A1:** Mean squared error (MSE) of ANN for different layers and units in training for 100 entries of the augmented dataset.

Layer	Unit = 2	Unit = 4	Unit = 8	Unit = 16
1	0.414	0.304	0.171	0.146
2	0.390	0.133	0.107	0.031
3	0.504	0.145	0.053	0.030
4	0.266	0.100	0.103	0.013
5	0.197	0.178	0.029	0.052

**Table A2:** MSE of ANN for different layers and units in training for 1000 entries of the augmented dataset.

Layer	Unit = 2	Unit = 4	Unit = 8	Unit = 16
1	0.412	0.412	0.147	0.140
2	0.290	0.109	0.057	0.038
3	0.502	0.183	0.040	0.054
4	0.306	0.084	0.052	0.032
5	0.189	0.157	0.052	0.012

**Table A3:** MSE of ANN for different layers and units in training for 10,000 entries of the augmented dataset.

Layer	Unit = 2	Unit = 4	Unit = 8	Unit = 16
1	0.503	0.189	0.188	0.132
2	0.389	0.094	0.026	0.030
3	0.177	0.147	0.147	0.011
4	0.190	0.071	0.042	0.052
5	0.190	0.071	0.043	0.028

**Table A4:** Variance of ANN for different layers and units in training for 100 entries of the augmented dataset.

Layer	Unit = 2	Unit = 4	Unit = 8	Unit = 16
1	0.577	0.683	0.801	0.836
2	0.590	0.853	0.874	0.947
3	0.481	0.834	0.939	0.962
4	0.734	0.889	0.876	0.963
5	0.788	0.803	0.940	0.923

**Table A5:** Variance of ANN for different layers and units in training for 1000 entries of the augmented dataset.

Layer	Unit = 2	Unit = 4	Unit = 8	Unit = 16
1	0.575	0.575	0.817	0.845
2	0.696	0.876	0.932	0.956
3	0.482	0.802	0.939	0.947
4	0.682	0.896	0.936	0.958
5	0.797	0.828	0.947	0.989

**Table A6:** Variance of ANN for different layers and units in training for 10,000 entries of the augmented dataset.

Layer	Unit = 2	Unit = 4	Unit = 8	Unit = 16
1	0.484	0.798	0.797	0.841
2	0.598	0.886	0.960	0.953
3	0.812	0.835	0.838	0.988
4	0.797	0.909	0.947	0.958
5	0.798	0.911	0.929	0.975
